# The Debate between the Human Microbiota and Immune System in Treating Aerodigestive and Digestive Tract Cancers: A Review

**DOI:** 10.3390/vaccines11030492

**Published:** 2023-02-21

**Authors:** Cátia Domingues, Cristiana Cabral, Ivana Jarak, Francisco Veiga, Marília Dourado, Ana Figueiras

**Affiliations:** 1 Laboratory of Drug Development and Technologies, Faculty of Pharmacy, University of Coimbra, 3000-548 Coimbra, Portugal; 2 LAQV-REQUIMTE, Laboratory of Drug Development and Technologies, Faculty of Pharmacy, University of Coimbra, 3000-548 Coimbra, Portugal; 3 Institute for Clinical and Biomedical Research (iCBR) Area of Environment Genetics and Oncobiology (CIMAGO), Faculty of Medicine, University of Coimbra, 3000-548 Coimbra, Portugal; 4 Center for Health Studies and Research of the University of Coimbra (CEISUC), Faculty of Medicine, University of Coimbra, 3000-548 Coimbra, Portugal; 5 Center for Studies and Development of Continuous and Palliative Care (CEDCCP), Faculty of Medicine, University of Coimbra, 3000-548 Coimbra, Portugal

**Keywords:** cancer, cancer therapy, chemotherapy, immune system, immunotherapy, microbiota, microbiome, nanotechnology

## Abstract

The human microbiota comprises a group of microorganisms co-existing in the human body. Unbalanced microbiota homeostasis may impact metabolic and immune system regulation, shrinking the edge between health and disease. Recently, the microbiota has been considered a prominent extrinsic/intrinsic element of cancer development and a promising milestone in the modulation of conventional cancer treatments. Particularly, the oral cavity represents a yin-and-yang target site for microorganisms that can promote human health or contribute to oral cancer development, such as *Fusobacterium nucleatum*. Moreover, *Helicobacter pylori* has also been implicated in esophageal and stomach cancers, and decreased butyrate-producing bacteria, such as *Lachnospiraceae* spp. and *Ruminococcaceae*, have demonstrated a protective role in the development of colorectal cancer. Interestingly, prebiotics, e.g., polyphenols, probiotics (*Faecalibacterium, Bifidobacterium, Lactobacillus*, and *Burkholderia*), postbiotics (inosine, butyrate, and propionate), and innovative nanomedicines can modulate antitumor immunity, circumventing resistance to conventional treatments and could complement existing therapies. Therefore, this manuscript delivers a holistic perspective on the interaction between human microbiota and cancer development and treatment, particularly in aerodigestive and digestive cancers, focusing on applying prebiotics, probiotics, and nanomedicines to overcome some challenges in treating cancer.

## 1. Introduction

The human microbiota refers to the aggregate of commensal microorganisms (such as bacteria, archaea, viruses, and small eukaryotes) which inhabit the human body and can establish symbiotic and pathogenic relations [1,2,3]. It is characterized by an enormous diversity, which can be described regarding its richness (number of species) or regularity (relative abundance of microorganisms of each species), being verified that the number of microorganisms inhabiting the human organism is of about 3.8 × 10^13^ [4]. Most studies focus on the intestinal microbiota since it represents the most extensive bacterial community. However, as the gastrointestinal tract begins in the oral cavity, the flora of this cavity should also be considered [5]. The heterogeneity of the human microbiota is not only interindividual. Actually, in the same person, the microbial composition can also differ depending on the site/target organ [6].

The human microbiota is developed over time and is determined by the interaction of multiple genetic variables, such as the type of delivery, the mother’s gestational age, diet, early exposure to antibiotics, as well as contact with the surrounding environment and lifestyles [7]. As a result, each individual develops a unique microbiota, presented in two separate domains, temporal and spatial [8]. 

**Figure 1:**
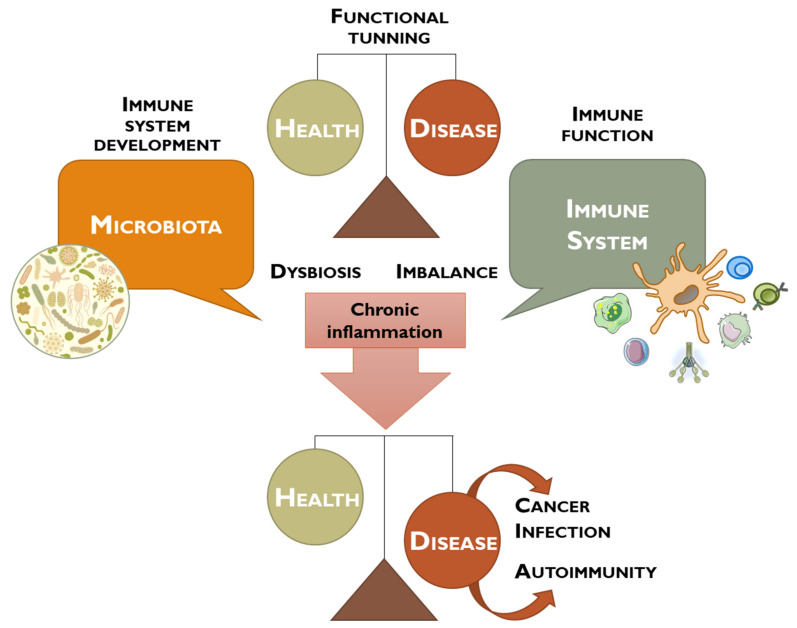
Schematic representation of the debate performed between microbiota and immune system to maintain the tight balance between health and disease. Dysbiosis contributes to an imbalance of the immune system, leading to chronic inflammation, and may promote the development of cancer, infection, or autoimmune diseases.

The term microbiome refers to the set of all microbes and their genetic elements, which is estimated to have 100 times more genes than the ones existing in the human body [3]. Thereby, the human microbiome, in combination with the host immune system, plays a critical role in balancing health and diseases in individuals, regulating physiological, neurological, and cognitive functions, as well as hematopoiesis, inflammation, and immunity (Figure 1) [2].

More recently, the interaction between human microbiota and cancer has been highlighted [9,10,11]. Indeed, it has been reported that different polymorphic populations of microorganisms, mainly bacteria, could impact cancer phenotypes, providing protective or harmful effects on cancer progression and responses to treatment [12].

Microorganisms and microbiota can contribute to the promotion or inhibition of carcinogenesis by regulating the balance between cell proliferation and death, the immune system, and the metabolic response to internal factors (produced by the host individual) or external factors (diet or drugs) [11]. It has been reported that microorganisms modulate 20% of carcinogenic events [10,11].

On one side, the microbiota can affect the response to conventional anticancer therapies, such as surgery, chemotherapy, radiation therapy, or immunotherapy. However, on the other side, the microbiota can also operate as a supportive cancer therapy [2,13].

Actually, the development of innovative microorganism-based therapies has attracted the scientific community for more than 100 years, with revolutionizing results since the formulation of Coley’s vaccine. Thus, the potential to modulate human microbiota has experienced profound significance and biotechnological advances, translated into an increase in the number of publications in the field, from 40 in 2000 to 6411 in 2022 (*Pubmed* database search from 2000-01-01 to 2022-11-07 on 7 November 2022), and global market trends. In fact, it is expected that the human microbiome market will increase from USD 209 million (2023) to USD 1370 million (2029), with a compound annual growth rate (CAGR) of 31.1% during the forecast period (2023–2029) [14].

Therefore, considering these trends, this review outlined the role of the microbiota in the development and progression of cancer, particularly in cancers of the aerodigestive tract and the gastrointestinal tract (GIT). Furthermore, the influence of microbiota in cancer treatment, specifically in surgery, chemotherapy, radiotherapy, and immunotherapy, was underscored. Later, some treatment strategies addressing the microbiota were proposed. Finally, some conclusions and future perspectives were provided. 

## 2. Human Microbiota, Dysbiosis, and Cancer

In recent decades, knowledge of the human body and its symbiosis with microorganisms has evolved exponentially and innovatively. The human being can be seen as a superorganism composed of vast and complex networking between human eucaryotic cells and non-human procaryotic cells. Trillions of bacteria colonize different parts of the human body, such as the skin, mouth, vagina and intestine, with the largest cluster being found in the GIT. 

The human intestinal microbiota represents all the microorganisms that can be found throughout the human GIT [15]. This microbiota mainly comprises obligate anaerobic microorganisms, with a predominance of *Firmicutes* and *Bacteroidetes phylum*, representing about 90% of the microbial system [15,16,17].

GIT not only functions as a food inlet and outlet but also as a part of the organism with several microenvironments, thus having a diverse ecosystem [5]. This variability is due to the distinct bacterial distribution along the GIT [15]. For example, the small intestine is abundant in *Firmicutes*, and the colon in *Bacteroidetes* [18]. Moreover, it has also been reported that the quantity of bacteria in each portion of the GIT is variable, with the colon presenting the most representative bacterial density [15,18].

Interestingly, most studies have reported that the maternal microbiota is the first contact of the newborn with microorganisms. More recently, some studies have proposed that bacterial colonization in the intestine of the fetus may start before birth because of the intrinsic intestinal microbiota of the progenitor, the placental circulation, and the amniotic fluid [7]. As previously mentioned, intestinal microbiota develops over time, and several determinants may influence its colonization, such as the type of delivery, the mother’s gestational age, and diet, among others [15,18]. Intestinal microbiota composition presents broad variations throughout the first year of life [18]. It is approximately by the age of three that the microbiota of children resembles that of the adult. The microbiota can remain stable for decades, although it may also be disturbed by several factors (either from the host itself or externally), leading to a change in its composition called dysbiosis [15].

While the intestine harbors many microorganisms, the oral cavity’s bacterial diversity is higher [5]. Whereas the intestinal microbiota is mainly constituted by the *Firmicutes* and *Bacteroidetes phyla* [15], the oral microbiota is rich in *Fusobacteria*, *Actinobacteria*, *Proteobacteria*, *Bacteroidetes*, and *Spirochaeta*, even though the *Streptococcus* genus is also present [5]. This diversity is possible due to the average temperature of 37 °C, which allows bacteria a favorable environment for their growth, as well as the saliva’s stable pH (6.5–7.5), which, in addition allows the appropriate hydration, the excellent growing environment, and also provides micronutrients’ transport to these microorganisms [19]. 

The microbiota and its host form a complex superorganism, conferring benefits to the host through the established symbiotic relation, such as regulation of the immune system and intervention in the metabolism. However, microbiota changes due to environmental variations (infection, diet, lifestyle, medication, and intestinal motility) may disrupt this symbiotic relation and promote disease [20,21]. 

Therefore, the microbiota plays an essential role in cellular homeostasis regulation, as changes in its composition (dysbiosis) may lead to immune system imbalance, eventually leading to an abnormal innate or acquired immune response [22].

**Figure 2:**
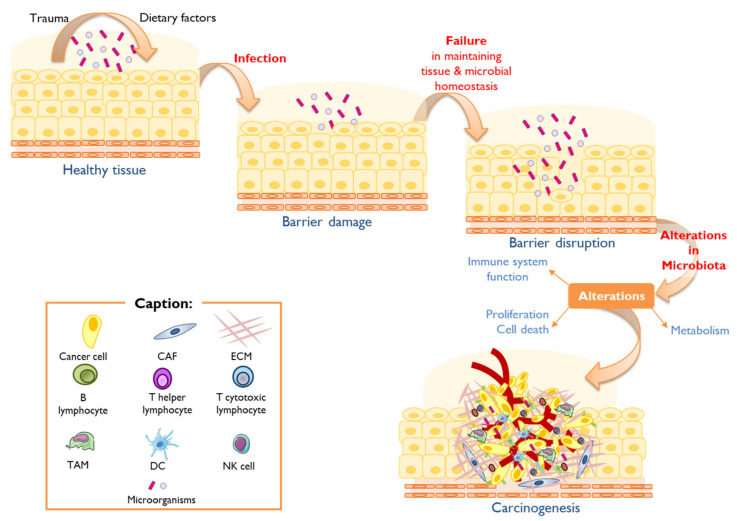
The contribution of microbiota on the solid tumor carcinogenesis process. Human body barriers are subject to constant environmental insult and injury. Trauma and dietary factors can contribute to the breach of the mucosal barriers, leading to infection. Generally, mucosal barrier damage is rapidly repaired, and tissue homeostasis is restored. However, decreased host resiliency contributes to persistent barrier damage, leading to its disruption and failure in homeostatic repair. In these settings, the microbiota may influence carcinogenesis by (i) altering host cell proliferation and death, (ii) perturbing immune system function, and (iii) influencing metabolism [11].

This dysbiosis is also often associated with allergy, metabolic dysregulation, inflammation, and cancer. When there is a dysbiosis, the interaction between epithelial cells and the microbiota changes, resulting in the alteration of the protective barriers and the failure of homeostatic cellular regulation, contributing to the carcinogenic process by the deregulation of proliferation pathways/cellular death, evasion of the immune system, and influence on the host’s metabolism (Figure 2) [2,11,22].

In fact, it has been estimated that 2.2 million cancers, e.g., solid, hematologic, and sarcomas, are attributable to infectious agents assigned as carcinogenic by the International Agency for Cancer Research (IACR) (Table 1) monographs program, corresponding to an age-standardized incidence rate (ASIR) of 25 cases per 100,000 people per year [23,24].

Most studies focus on the intestinal microbiota since it represents the broader bacterial community, with evident results in its correlation with different gastrointestinal pathologies. However, the gastrointestinal system begins in the oral cavity, which means the specific flora of this cavity must also be investigated [5]. The oral cavity is located in the aerodigestive system with an abundance of 772 species of prokaryotes [62].

The following sections will mainly be focused on the role of microbiota in aerodigestive and digestive cancers, particularly oral, esophageal, stomach, and colon.

### 2.1. Oral Cancer

**Figure 3:**
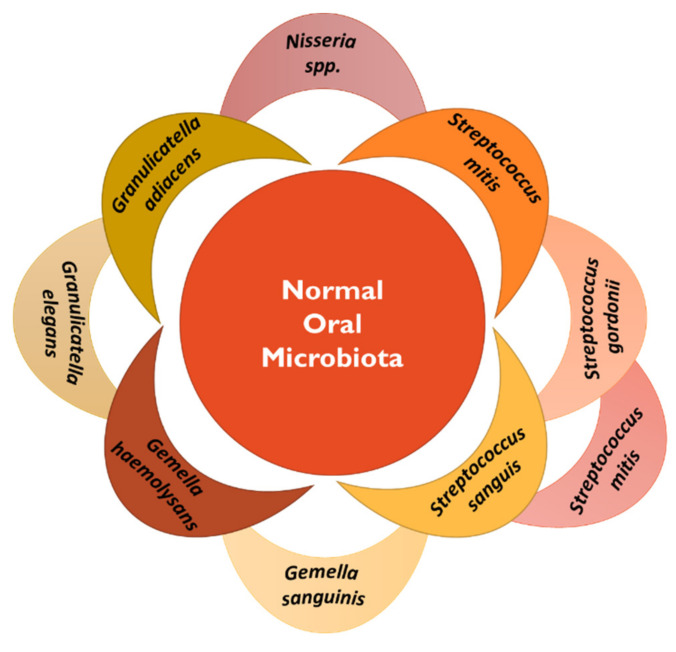
Summary of the microbial species present in the normal oral microbiota [64].

Oral microbiota has been widely studied since the availability of new-generation sequencing modalities. Based on these advancements, oral microbiota composition has been found to comprise ca. 700 taxa species [63]. The most common microbial species presented in the normal bacteria flora of the oral cavity are summarized in Figure 3 [64]. The presence of these communities is helpful in the maintenance of oral health. Therefore, some studies have reported the association of oral dysbiosis with the development of some pathologies, particularly oral cancer [65].

Oral cancer accounts for 40% of all head and neck cancers. It is a multifactorial and heterogeneous disease with a high morbidity and mortality rate, mainly due to its late diagnosis. The most common histological type is oral squamous cell carcinoma (OSCC), corresponding to about 90% of cases. OSCC results from the interaction between genetic/epigenetic events, environmental factors, hygiene habits, alcohol consumption, and smoking [66]. Nonetheless, 15% of oral cancer diagnoses are not directly linked with such risk factors and may be associated with other pathologies such as dental caries, plaque, gingivitis, and periodontitis [5,67].

For instance, regarding periodontitis, one of the primary pathogens is the *Porphyromonas gingivalis*, which can invade eukaryotic cells through different virulence mechanisms, such as adhesion to epithelial cells or inhibition of the immune system [5]. This immune system evasion may be a lever for developing oral cancer or other types of cancer, and periodontitis may be indicated as a possible risk factor for its development. However, further studies are required [68].

The so-called bacterial biofilm, which covers the surface of the oral cavity, can contribute to the development of tumor microenvironment when both qualitative and quantitative changes occur in the bacteria environment. Several studies have found that the *Fusobacterium* genus was involved in the OSCC and has demonstrated a steady diagnostic ability. Smoking may affect the biofilm structure, resulting in unstable colonization, thus increasing the individual susceptibility to bacterial infections by deregulation of innate and adaptive immune responses. Börnigen et al. have demonstrated that the abundance of *Firmicutes* (*Lactobacillus*, *Veillonella*, and *Streptococcus*), *Actinobacteria* (*Bifidobacterium* and *Atopobium*), *Proteobacteria* (*Neisseria*), as well as *Bacteroidetes* (*Prevotella*) undergoes alterations in patients with smoking habits [69].

Human papillomavirus (HPV), a risk factor associated with a great variety of cancers, such as cervical and head and neck cancer, has been associated with the malignant transformation of oral keratinocytes, with *Streptococcus* spp. as a cofactor in such modifications [69]. The oral microbiota can be amended during the different antineoplastic treatments. For example, in radiotherapy, there are alterations in the antibacterial properties of saliva, with consequence alteration in the oral microbiome and decrease in the pH, thus increasing the pathogenic potential in the oral cavity [5]. As a precautionary measure, patients should establish and maintain adequate oral hygiene before and after treatment [5].

Identifying and quantifying the microorganisms through sequencing methods, such as metagenomics, allows a better understanding and evaluation of the microbial community [8,70]. Associating these studies with the assessment of the environmental factors which affect the microbiome could be a promising approach in the early diagnosis of oral cancer [69].

Although the oral cavity is continuously subjected to food and fluid intake as well as other external changes, it remains relatively stable over time in healthy people. Therefore, several studies have evaluated the oral bacterial profile’s connection to cancer diagnosis [71]. Hence, the microbiota provides an ideal source for discovering biomarkers due to low inter- and intra-biological variations in contrast to other biomarkers [19]. Considering that saliva is an abundant and accessible biofluid, it can be used as a non-invasive sample and is quite promising for the detection of biomarkers and for monitoring oral carcinogenesis and response to therapy [5,72]. A study conducted by Schmidt et al. (2014) investigated the oral microbiome of five patients with oral cancer and eight patients in the pre-neoplastic stage, using the 16s ribosomal RNA (rRNA) gene sequencing. This study demonstrated a significant decrease in the abundance of *Firmicutes* and *Actinobacteria* in patients with oral cancer [19].

Epithelial-mesenchymal transition (EMT) is a complex process that has been considered a hallmark of oral cancer [66]. Periodontal pathogens, such as *Fusobacterium nucleatum* and *Porphyromonas gingivalis*, have been associated with the promotion of EMT in primary oral keratinocytes [73]. Moreover, *Fusobacterium nucleatum* has been indicated as a potential trigger of lncRNA/miR4435-2HG/miR-296-5p/Akt2/SNAI1 and with the up-regulation of N-cadherin, Vimentin, and Snail Transcription Repressor 1, culminating in a more pronounced mesenchymal phenotype and leading to the EMT behavior [74]. These results may indicate that periodontal pathogens are involved in the promotion of EMT and may play a role in malignant transformation.

### 2.2. Esophageal Cancer

Esophageal cancer (EC) is ranked as the tenth most occurring cancer, with more than 604,000 new cases, and the sixth more lethal with more than 544,000 deaths, in 2020 [75].

The presence of microorganisms in the esophagus is crucial as they regulate fundamental processes of esophageal physiology, namely metabolism and immune maturation. Therefore, changes in their relative abundance have been implicated in the development of esophageal diseases [75].

Chronic inflammation in the esophagus terminal area, caused by gastroesophageal reflux, is closely related to the development of esophageal adenocarcinoma (EA). The general pathophysiology process can be described as gastroesophageal reflux disease–Barrett’s esophagus–esophageal adenocarcinoma (GERD-BE-EA) [9].

Several investigators suggest that EA morbidity may be associated with the use of antibiotics since this exposure induces alteration of the esophageal microbiota, leading to the development of a carcinogenic process [9].

Table 2 summarizes some studies conducted to provide insight into the role of microbiota in developing esophageal pathologies.

Other studies also suggest that *Helicobacter pylori* (*H. pylori*) may also play a role in GERD and EA. In the 1990s, *H. pylori* was first identified by the WHO as a carcinogen associated with stomach cancer. However, some studies claim that *H. pylori* infection may play a protective role in developing GERD and EA, possibly because it affects the pH of the stomach and promotes acid reflux [9]. However, the influence of *H. pylori* in the etiopathogenesis of EA remains uncertain and controversial [9].

### 2.3. Stomach Cancer

Stomach cancer is the fourth most common cancer in the world [71] and is often associated with inflammation [83], namely by *H. pylori* infection [84]. *H. pylori* colonizes the gastric mucosa of about 50% of the global population [22,85]. The presence of *H. pylori* causes inflammation and loss of acid-producing parietal cells, which may lead to gastric atrophy and the induction of carcinogenesis. Cancer progression occurs in 1 to 3% of the individuals infected with *H. pylori* [86]. It may be due to the genetic diversity of *H. pylori*, differences in host responses, and environmental factors that can determine the disease’s prevalence and severity [9,84].

Several virulence factors have been anticipated for *H. pylori* infections, such as vacuolating cytotoxin A (vacA) and cytotoxin-associated gene A (cagA). These are produced by *H. pylori* and induce alterations of the gastric epithelium by disturbing the cell cycle and proliferation, leading to cell death, and compromising the normal function of the immune system. The host cannot eliminate *H. pylori*, which may lead to chronic inflammation, contribute to genomic instability, and subsequently to carcinogenesis [9,71].

Other types of bacteria have been associated with the development of gastric cancer, as previously reviewed [87,88]. Briefly, *Propionibacterium acnes (P. acnes)* and *Prevotella copri (P. copri)* have been reported to be more abundant in samples collected from patients with gastric cancer than in healthy controls, being considered a risk factor for disease development in a Korean population-based study. On the other hand, *Lactococcus lactis* seems to play a protective role in the development of gastric cancer [89]. Moreover, *Fusobacterium* sp. Have been reported to be positively correlated with tumor-infiltrating lymphocytes, affecting phenotypic characteristics and metabolic function in gastric cancer [90].

*Streptococcus*, *Lactobacillus*, *Veillonella*, and *Provotella* genera have also been found to be significantly abundant in samples from patients with gastric cancer [91,92]. Increased levels of *Lactobacillus*, a lactic acid bacterium with probiotic activities, have been reported in intestinal metaplasia or gastric cancer [93]. 

The role of the extragastric microbiome, particularly the role of enterohepatic *Helicobacter sp.,* has also been emphasized in attenuating or promoting gastric pathology using C57BL/6 mice models [87]. Moreover, oral microbiota have also been associated with the development of gastric pathology, namely gastric cancer [94]. Actually, a higher relative abundance of oral-related bacteria, e.g., *Leptotrichia*, *Fusobacterium*, *Haemophilus*, *Veillonella*, and *Campylobacter*, have been accounted for in patients with gastric cancer [95]. Furthermore, commensals or opportunistic pathogens from the genera *Neisseria, Alloprevotella*, and *Aggregatibacter*, species *Streptococcus_mitis_oralis pneumoniae* and strain *Porphyromonas_endodontalis.t_GCF_000174815* that are generally present in the oral cavity have also been identified in samples from patients with gastric cancer [96].

### 2.4. Colorectal Cancer

The connection between intestinal microbiota and colorectal cancer (CRC) development has recently been reviewed [97,98,99].

In brief, in colorectal adenomas and CRC, the microbiota is typified by the imbalance between the relative abundance of potentially pathogenic bacteria, such as *Pseudomonas*, *Helicobacter*, and *Acinetobacter* [100]. On the other hand, decreased butyrate-producing bacteria, such as *Lachnospiraceae* spp. and *Ruminococcaceae*, have provided information on the importance of metabolic regulation of CRC and demonstrated a protective role on its development by the enrichment of a fiber-rich diet [101,102].

*Bacteroides massiliensis*, *Bacteroides ovatus*, *Bacteroides vulgatus* e *Bacteroides fragilis*, and *Escherichia coli* have been associated with the malignant transformation of advanced colorectal adenoma into CRC [103]. *Fusobacterium nucleatum*, an existing microorganism in the oral microbiota, has also been implicated in CRC by modulating the tumor-immune microenvironment [104,105]. Considering this, *Fusobacterium nucleatum* has been studied as a potential biomarker for CRC development [106]. However, additional studies are required.

## 3. The Interplay between Microbiota and Cancer Treatment

The impact of microbiota, especially the gut microbiota, on the modulation of cancer treatment and the susceptibility to side effects has been addressed [24,107] Pharmacomicrobiomics has arisen to exploit drug-microbiota interactions in anticancer therapies [108,109].

**Figure 4:**
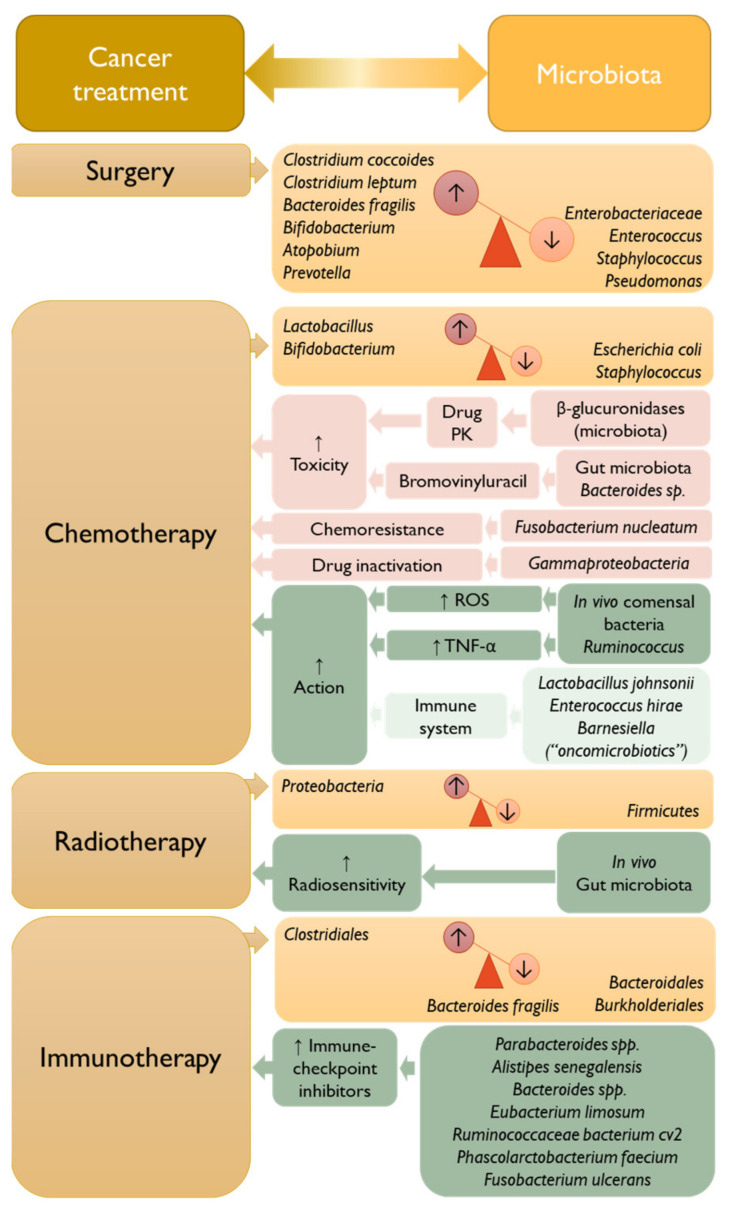
Overview of the common anticancer treatments and their influence on the microbiome and vice-versa [110]. PK, pharmacokinetics; ROS, reactive oxygen species; TNF-α, tumor necrosis factor—alpha.

The following topics will address the interplay of microbiota and cancer treatments, e.g., surgery, chemotherapy, radiotherapy, and immunotherapy (Figure 4) [84,107,109,110], addressing aerodigestive and digestive cancers.

### 3.1. Surgery

Generally, surgery is considered one of the standard treatments for localized solid tumors without metastasis [111,112,113]. In these cases, the influence of microbiota on the disease outcome is mainly recognized in CRC pathologies [110,114], and also in oral cancer from the tongue [115].

In a study conducted by Ohigashi et al. [116] it was observed that the total counts of important microorganisms that regulate microbiota homeostasis were altered after CRC surgery. Indeed, the total counts of obligate aerobes, such as *Clostridium coccoides*, *Clostridium leptum*, *Bacteroides fragilis*, *Bifidobacterium*, *Atopobium*, and *Prevotella,* were diminished. On the contrary, the total counts of facultative anaerobes, e.g., *Enterobacteriaceae*, *Enterococcus*, and *Staphylococcus*, and the aerobe *Pseudomonas* were significantly increased after surgery. These alterations may induce postoperative anastomotic and infectious complications and impact treatment outcomes [116].

Therefore, prophylactic antibiotic therapy has been proposed to overcome some of these undesirable outcomes. However, its application is not currently consensual and more studies are needed [117].

### 3.2. Chemotherapy

Cytostatic medicines are classified according to their action mechanisms, such as alkylating agents, heavy metals, antimetabolites, and topoisomerase inhibitors. Most of these exhibit their activity at the deoxyribonucleic acid (DNA) level, either directly or during replication, and may also affect other cellular components, such as mitochondria or membranes [2].

It is known that more than 40 medicines are shown to be metabolized by the intestinal microbiota, but only a few are affected. Moreover, enteral or parenteral administration of these active pharmaceutical ingredients (API) may induce dysbiosis at the GIT level [2,84]. 

#### 3.2.1. Platinum-Based Anticancer Therapy—Oxaliplatin and Cisplatin

Platinum compounds induce cytotoxic effects by mediating reactive oxygen species (ROS) production, leading to tumor cell death [2,118]. However, oxaliplatin also mediates cell death by immunogenic cancer cell death in contrast to cisplatin.

In addition to killing tumor cells, platinum drugs are associated with undesirable secondary adverse events, namely, intestinal toxicity, nephrotoxicity, loss of the integrity of the blood–brain barrier (BBB), and ototoxicity.

In studies conducted in mice xenografts, including MC38 colon carcinoma-derived models, the antitumor effect of oxaliplatin or cisplatin decreased dramatically in the antibiotics-treated group compared with the germ-free group, indicating that an intact commensal microbiota is required for effective cancer treatment [119].

Moreover, in a murine model of intestinal mucositis in the context of the antineoplastic agent cisplatin, the 16S rRNA sequencing analysis of fecal DNA indicated that cisplatin induces dysbiosis with a significant increase in *Bacteroidaceae* and *Erysipelotrichaceae* families and *Bacteroides uniformis*, with a decrease in *Ruminococcus gnavus* [120].

On the other hand, the treatment with oxaliplatin induce dysbiosis in the murine colon, with a significant reduction in genus *Parabacteroides* and *Prevotella_1_* and increases in *Prevotella_2_* and *Odoribacter* in the murine colon [121]. However, the mechanisms related to gastrointestinal dysfunction driven by oxaliplatin seem not to be associated with inflammatory enteric neuropathy. Still, more research is required to understand the significance of this correlation [121].

#### 3.2.2. Alkylating Agents—Cyclophosphamide

Cyclophosphamide (CTX) is an alkylating chemotherapeutic agent used in the treatment of patients with advanced cancer, including gastric cancer [122]. CTX mainly acts by the induction of immunological cell death by affecting the immunosuppressive environment of the tumor, inducing a reduction of Treg cells, an increase in T helper (Th)-1 cell differentiation [109] and Th-7 cells, and the conversion of naive T CD4^+^ cells into Th17 cells [118]. In addition to that, CTX induces an adaptive antitumor immune response [2].

Furthermore, in mice tumor xenografts, it was reported that the administration of CTX induces the translocation of bacteria, such as *Enterococcus hirae* and *Lactobacillus johnsonii*, from the gut to secondary lymph nodes, leading to the accumulation of Th-17 and Th-1 cells that are essential for the anticancer immune response of CTX [123]. Furthermore, *Enterococcus hirae* is also responsible for restoring the action of CTX in antibiotic-treated mice [124]. *Barnesiella intestinihominis* accumulates in the colon and stimulates the intratumoral infiltration of IFN-γ-producing γδ-T cells [124,125].

#### 3.2.3. Irinotecan

Irinotecan (also known as CPT11) is a topoisomerase I inhibitor that blocks DNA replication preferentially in rapidly dividing cells [126]. Irinotecan is administered intravenously to treat a variety of solid tumors, particularly advanced CRC [127].

Irinotecan is biotransformed into its active metabolite ethyl-10-hydroxy-camptothecin (SN/38) at the hepatic level and small intestine tissue carboxylesterase, being cleared in the liver by host UDP-glucuronosyltransferases into inactive SN-38-G and secreted into the gut. Once in the gut, SN-38-G can be reconverted into active SN-38 by the β-glucuronidases, inducing gastrointestinal toxicity (nausea, vomiting, diarrhea) [2,126]. The action and toxic mechanisms of irinotecan have been described to be influenced by the microbiota, namely through: microbial ecocline, catalysis of microbial enzymes, and immunoregulation, which is essential for the maintenance of intestinal homeostasis [126].

In vivo data have reported that the administration of irinotecan increases the production of β-glucuronidase bacteria, such as *Escherichia coli* [128], *Staphylococcus* spp., and *Clostridium* spp., and reduces the number of non-producing β-glucuronidase bacteria, such as *Bifidobacterium* spp. and *Lactobacillus* spp. [129,130].

Irinotecan may also induce dysbiosis by increasing the abundance of *Clostridium* cluster XI and *Enterobacteriaceae* in the proximal colon, which may lead to inflammation or changes in the proportion of bacteria expressing β-glucuronidase, as reported in tumor-bearing rats [131]. In the human colonic ecosystem, *Firmicutes* phylum, particularly *Clostridium* clusters XIVa and IV, has been found to present the highest β-glucuronidase activity [132,133].

Recently, Lian et al. [134] have proposed that the intestinal microbiota may not constitute a central puzzle piece in mediating gastrointestinal alterations, which is controversial and may not be described by other studies [127].

Moreover, Toll-like receptor (TLR) pathways are also implicated in irinotecan-induced gastrointestinal mucositis and pain, mainly by the activation of TCR4 signaling [135,136]. The underlying mechanism may be mediated by the interaction of irinotecan with the complex TCR4/myeloid differential protein-2 (MD-2) that explicitly recognizes lipopolysaccharides on the cell wall of Gram-negative bacteria, leading to the activation of the innate immune response [137,138]. Therefore, the potential direct binding of SN-38 to MD-2 and its TLR4 complex may improve the pharmacological control of mucositis. However, more studies are required, as IRT-induced delayed diarrhea is a complex event with multiple players such as NFκB, TLR4, Aquaporin-3 (AQP3) water channel, and the transient receptor potential cation channel A1 (TRPA1) receptor, among others [127]. Moreover, off-target immunological effects may also be critical orchestrators of irinotecan-toxicity-mediated mechanisms [139].

Therefore, using small molecule drugs, plant extracts, or probiotics has been proposed to limit the excessive formation of SN-38 in the intestine [140]. For example, supplementation with the *Bifidobacterium* probiotic can attenuate intestinal injury caused by irinotecan in mice [141].

### 3.3. Radiotherapy

Radiotherapy is genotoxic for tumor cells, representing one of the most commonly used treatments for localized tumors, including cancers from the aerodigestive and digestive tract [142,143,144]. The effects of radiation are complex, activating immunostimulatory and immunosuppressive responses, which may be insufficient to trigger a protective anticancer immune response. Ionizing radiation can induce effects on healthy non-irradiated cells and lead to inflammatory and immune reactivation, thus releasing signs of stress [2,145].

**Figure 5:**
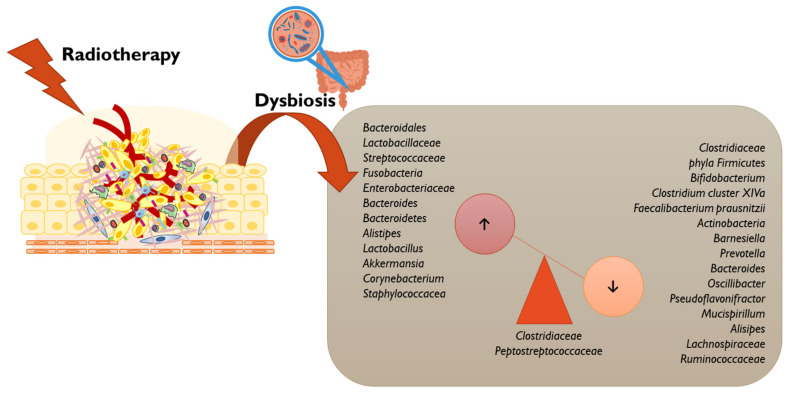
Radiotherapy may compromise the gut microbiota homeostasis by the imbalance of some genera, as depicted [146,148,149,150,151,152].

The intestinal microbiota has been shown to affect the immune response induced by immunogenic cell death in chemotherapy, and it may also play a role in the immunostimulatory effects of radiotherapy [2]. The role of radiotherapy in microbiota homeostasis has been previously reviewed (Figure 5) [146,147].

Radiotherapy induces apoptosis of the intestinal crypts, breaking the intestinal barrier and leading to changes in the microbiota composition. These changes allow pathobionts to access the intestinal immune system, leading to inflammation of the intestine. Moreover, radiotherapy may also contribute to oral mucositis and diarrhea [2].

The effects of gut microbiome on cancer radiotherapy have also been addressed in preclinical studies [147]. A study carried out with mice revealed that radiotherapy-mediated gastrointestinal damage can be reduced by blocking the signaling of TLR3. TLR3 are critical receptors involved in regulating radiation-mediated intestinal toxicity. Mice with a deficiency in these receptors have shown a higher survival rate when exposed to ionizing radiation and lower intestinal toxicity when compared with control mice (without TLR3 deficiency) [153,154].

Some clinical studies have demonstrated that the use of probiotics may be beneficial in preventing radiation-induced enteropathy. Formulations containing *Lactobacillus acidophilus*, *Bifidobacterium bifidum,* and *Lactobacillus casei*, or with *Bifidobacterium*, *Lactobacillus*, and *Streptococcus* spp. were considered protective against intestinal toxicity induced by radiotherapy by significantly reducing the incidence of severe diarrhea [152,155]. In the study by Manichanh et al. it was proved that the intestinal microbiota undergoes significant changes after pelvic radiation in patients with neoplasms in the abdominal region. In fact, it was observed that patients who received radiotherapy and developed diarrhea as a side effect presented an increase in *Actinobacteria* and *Bacilli* and a decrease in *Clostridia* [156].

An improved understanding of the effect of non-targeted/non-specific radiation and its regulation by the commensal microbiota or its therapeutic manipulation may be a promising approach for enhancing therapeutic efficacy and reducing the secondary toxicity of radiotherapy [147].

### 3.4. Immunotherapy

Despite advancements in cancer treatment, particularly regarding chemo- and radiotherapy, patients acquire resistance to conventional therapies with frequent relapses and high side effects [2]. Therefore, immunotherapy has recently emerged as a new therapeutic approach with promising results in cancer treatment and reduced side effects, particularly in oral [157], esophageal [158], stomach [159], and colorectal cancers [160].

Nonetheless, some limitations in cancer immunotherapy have been reported, namely due to tumor heterogeneity, which influences the therapeutic efficacy and variability of the immune response in different patients [2]. Indeed, the effectiveness of immune checkpoint blockers, such as CpG-oligodeoxynucleotide, anti-CTLA-4, and anti-programmed death-ligand 1 (PD-L1), have appeared to be dependent on the intestinal microbiome, which intimately interacts with the immune system [84,161,162,163].

#### 3.4.1. CpG Oligodeoxynucleotide

The unmethylated cytosine-phosphate-guanosine oligodeoxynucleotide (CpG-ODNs) are potent agonists of the TLR-9. These receptors are essential transmembrane type I proteins that are part of the innate immune system [164]. CpG-ODNs induces the secretion of proinflammatory cytokines from myeloid cells, such as TNF and Interleukine (IL)-12, leading to necrosis and the repositioning of tumor-infiltrating macrophages and dendritic cells from an anti-inflammatory to a proinflammatory state, which elicits an antigen-specific adaptive T cell antitumor immunity, culminating in tumoral eradication [165,166,167]. Conversely, in germ-free mice or mice treated with a cocktail of non-absorbable antibiotics, e.g., vancomycin, neomycin and imipenem, the CpG-ODN treatment was limited, and tumoral development was observed. These studies suggest that deficient microbiota decreases the efficacy of CpG-ODN [119].

CpG-ODNs lead to the secretion of TNF-α in the tumor microenvironment [13]. This TNF-α production is somewhat related to the existence of bacterial genera, which exist in the microbiota at the time of treatment. For instance, the presence of Gram-negative *Alistipes* and Gram-positive *Ruminococcus* is related to TNF-α production, while *Lactobacillus*, including *Lactobacillus murinum*, *Lactobacillus intestinalis,* and *Lactobacillus fermentum*, are negatively related to the production of TNF-α [2]. In germ-free or antibiotic-treated mice, the production of TNF-α is diminished, therefore providing an ineffective response to therapy [119]. However, if the intestinal microbiota is re-colonized with *Alistipes shahii*, the ability to produce TNF-α is restored, which is not observed with *Lactobacillus fermentum* [168].

These results indicate that an exhaustion of the intestinal microbiota results in decreased response to CpG-ODNs treatment, although its recolonization may modulate the response to immunotherapy [2,11].

#### 3.4.2. Anti-CTLA4

After blocking the CTLA-4 receptor (Cytotoxic T-lymphocyte antigen 4), intraepithelial lymphocytes damage the mucosa of the ileum and colon [156], altering the composition of intestinal and fecal microbiota [2]. Antitumor effects of this therapy also depend on the intestinal microbiota, especially of *Bacteroides fragilis* presence. When mouse feeding is rich in *Bacteroides fragilis* and *Burkholderia cepacia*, the anti-CTLA4 response is improved, in addition to a significant decrease in intestinal damage associated with the antitumor response [2,71].

Moreover, the immunotherapeutic effect of CTLA-4 blockade depends on distinct *Bacteroides sp*. In fact, in a study performed by Vétizou et al. [169] in mice and patients, specific responses regarding T cells to *B. thetaiotaomicron* or *B. fragilis* were associated with the efficacy of anti-CTLA-4 treatments. These antitumoral effects were not observed in antibiotic-treated or germ-free mice but were circumvented by gavage with *B. fragilis* [169].

Besides that, gut microbial metabolites can trigger the modulation of immune responses in the gut. Along the colon, short-chain fatty acids (SCFA) are produced in large amounts by the bacterial fermentation of dietary fiber. Therefore, the levels of SCFA may influence the efficacy of anti-CTLA-4 blocking mAbs [170].

#### 3.4.3. Anti-PDL1

The programmed death-1 (PD-1) is an essential cell surface receptor that works as a checkpoint and plays a crucial role in controlling T cell exhaustion. The binding of PD-1 to its ligand, PD-L1, activates downstream signaling pathways blocking T cell activation. Abnormally high expression of PD-L1 in tumor cells and antigen-presenting cells in the TME are key orchestrators of tumor immune escape, constituting the main focus of anti-PD-1/PD-L1 antibodies [171,172].

The human gut microbiota has been associated with clinical outcomes to anti-PD-1/PD-L1 immunotherapy in melanoma, non-small cell lung cancer, renal cell carcinoma, and stomach cancer [173,174]. Briefly, the enrichment of *Akkermansiacea muciniphila* in anti-PD-L1 responders is associated with the increase activation of DCs, leading to the secretion of IL-12, promoting the trafficking of CD4^+^ CCR9^+^ memory T cell and CD4^+^ CXCR3^+^ T cells from mesentery lymph nodes (mLNs) to tumor-draining lymph nodes (dLNs), culminating in the increase in antitumor response. Moreover, the presence of *Ruminococcaceae, Clostridales,* and *Feacalibacterium* in the GI tract has been associated on one side with the rise of the CD4^+^ and CD8^+^ T cell ratio and on the other side with the downregulation of regulatory T cells and myeloid-derived suppressor cells (MDSCs). Furthermore, anti-PD1/PD-L1 therapy can also be modulated by bacteria-associated metabolites through the peripheral differentiation of Th1 cells, potentiating DCs function and decreasing circulating T_reg_ cells, contributing to a reinforcement of the immune system [175].

**Figure 6:**
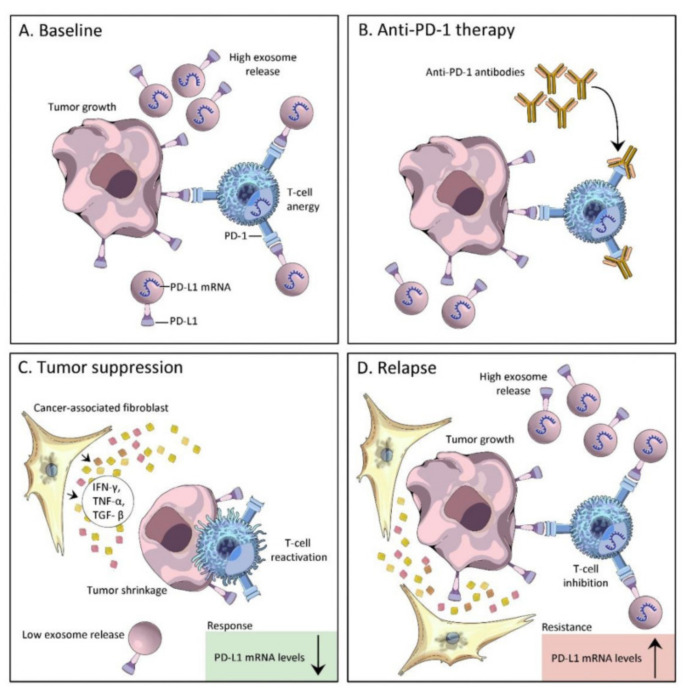
Exosomal PD-L1 correlates with tumor response and resistance to anti-PD1 therapy: (**A**) Tumor cell-derived extracellular vesicles cause immune suppression by the direct engagement of PD-1 on T cells; (**B**) PD-L1/PD-1 interaction is blocked by the presence of anti-PD-1 monoclonal antibody; (**C**) Tumor suppression—PD-L1 expression levels in exosomes are inversely related to the tumor’s response to immunotherapy. PD-L1 mRNA levels significantly declined from the start of treatment in patients with complete and partial responses to anti-PD-1therapy, characterized by low exosome release, T cell reactivation, and tumor shrinkage; (**D**) Tumor relapse—PD-L1 expression levels in exosomes are directly related to tumor resistance to immunotherapy. PD-L1 mRNA significantly increased in patients with a tumor relapse, characterized by increased exosome release, T cell inhibition, and tumor growth. Downwards arrow—decreased, upwards arrow—increased. Reprinted from [176] under a CC By license.

Although the role of bacterial extracellular vesicles (bEVs) in cancer pathophysiology is not yet fully processed, they present the ability to cross physiological barriers, assemble around the tumor cells, and lead to alterations in the TME, which may impact anti-PD-1/PD-L1 therapies (Figure 6) [176].

Recently, Li et al. [177] developed engineered bacterial outer membrane vesicles (OMVs) decorated with the ectodomain of the immune checkpoint PD-1 with promising improvements in anti-tumor immune responses, with reduction in tumor volume in CT26 Balb-c mice carrying tumors derived from murine CT26 colorectal cancer cells. 

## 4. The Microbiota as Target Therapy

Certain medicines, in particular antibiotics, may induce intestinal dysbiosis [15]. However, on the other hand, pre- and probiotics may help maintain the intestinal microbiota and interfere with the effectiveness of anticancer treatment [17].

### 4.1. Prebiotics

Prebiotics are non-digestible but fermentable polysaccharides that can selectively stimulate the growth, activity, or both of various bacterial species present in the colon, in ways that clearly maintain or promote health and prevent diseases [178]. The majority of prebiotics are oligosaccharide carbohydrates, such as fructans, galacto-oligosaccharides, glucose-derived oligosaccharides such as starch, and other oligosaccharides (pectic oligosaccharide). More recently, non-carbohydrate oligosaccharides have been recommended to be classified as prebiotic, namely the cocoa-derived flavanols [179].

Prebiotics protect the human organism by different mechanisms, defending against pathogens, promoting immune modulation, mineral absorption, bowel function, metabolic effects, and satiation. Liong et al. have previously reviewed the role of prebiotics in colon cancer [180].

Glucans and fructans have generally been classified as beneficial for humans, and evidence has arisen for oligomers of mannose, glucose, xylose, pectin, starches, human milk, and polyphenols [181]. Indeed, the use of prebiotics or dietary compounds such as polyphenols that can exert prebiotic effects has been studied. Polyphenols derived from green, oolong, and black tea have also been indicated to increase the in vitro abundance of *Bifidobacterium* spp. and *Lactobacillus*, and inhibit the proliferation of *Bacteroides, Prevotella*, and *Clostridium histolyticum* [182].

Interestingly, inulin and mucin induce changes in gut microbiota taxa and promote anti-tumor immunity. Specifically, inulin enhances the efficacy of a mitogen-activated protein kinase (MEK) inhibitor against melanoma with a delay in drug resistance [183].

Prebiotics such as bilberry anthocyanins have been reported to potentiate the effects of anti–PD-L1 therapy in a murine colon cancer model through the increase in the infiltration of CD8^+^ T cells and monitoring of the tumor growth [184].

Hence, reasonable dietary control through the ingestion of fiber and prebiotics may be a promising approach to decreasing cancer incidence and modulating anticancer therapy [9].

As the main target bacteria of prebiotics are *Bifidobacteria* and *Lactobacilli,* amplifying the performance spectrum, possibly for butyrate-dependent bacteria, could be a promising approach to overcome some anticancer drawbacks [185].

### 4.2. Probiotics

Probiotics define the population of bacteria that reside in the intestine and can have several beneficial effects on the host. The most common types of probiotics are lactic acid bacteria (LAB), mainly the genera *Lactobacillus* and *Bifidobacterium*, but also include *Enterococcus*, *Streptococcus*, and *Leuconostoc* [17]. Suggested mechanisms comprise inhibition of pathogen adhesion to the intestinal mucosa, stabilization of the microbial community, or improvement of mucosal integrity and barrier function [186]. The proportion of microorganisms may be an indicator of the individual’s health status, such as the proportion of *Bifidobacterium* to *Escherichia* (B/E)—in the case of a patient with CRC, the number of *Bifidobacterium* decreases drastically, while that of *Escherichia* increases [186]. Several studies show that oral administration of *Bifidobacterium* alone can influence the immune response against tumors in various mice models [186]. Treatment with *Lactobacillus rhamnosus* as a prophylactic measure could reduce the incidence and multiplicity of colon tumors by inducing cellular apoptosis and inhibiting inflammation. On the other hand, the administration of Lactobacilli in mice showed regular expression of several TLRs, decreasing tumor occurrence [17].

Studies carried out in melanoma animals have shown that probiotics can improve immunotherapy using immune checkpoint blockers. Through other studies, it has also been possible to conclude that germ-free mice exhibit less gastrointestinal damage and tolerate higher doses of irinotecan than control mice [6]. Therefore, the maintenance of the human microbiota plays a crucial role in preventing the development of the carcinogenic process. It is, however, necessary to consider that anticancer treatments may interfere with the normal integrity of the microbiota.

Probiotics such as *Faecalibacterium, Bifidobacterium, Enterococcus hirae, Akkermansia muciniphila, Lactobacillus*, and *Burkholderia* could modulate the diversity of the gut microbiota and enhance antitumor immunity [187].

These results suggest that the microbiota may be a therapeutic target for the success of conventional and novel cancer treatments [84].

More recently, the application of the so-called postbiotics has been addressed [187]. Postbiotics are classified as non-viable bacterial products or functional bioactive compounds resulting from the fermentation of probiotic microorganisms aiming to confer health on the host [188]. For instance, inosine, butyrate, or propionate could modulate the efficacy of immune checkpoint inhibitors [170,189]. Moreover, the use of postbiotic derived from *Lactobacillus* could exert antitumoral activity against HT-29 human colon cancer cells [190].

### 4.3. Fecal Microbiota Transplantation (FMT)

In 1958, Eiseman and colleagues used fecal microbiota transplantation (FMT) to treat *Clostridium difficile* infection, aiming to transplant functional microbiota from healthy individuals into the gastrointestinal tract of patients to rebuild normally functioning intestinal microbiota [110].

The impact of FMT has been studied in many pathologies, including cancer [191,192].

Gopalakrishnan et al. [193] divided melanoma patients receiving anti-PD-1 immunotherapy into responders and non-responders in order to verify if the gut microbiome diversity and composition influence the response to treatment. They observed that *Faecalibacterium* was more abundant in FMT responders, while FMT non-responders had higher abundances of *Bacteroides thetaiotaomicron*, *E. coli*, and *Anaerotruncus colihominis* [193]. FMT has also presented promising applications in patients with refractory metastatic melanoma [194] as well as in patients with gastrointestinal cancers [195]. 

Although FMT presented promising results, only 30% of patients have benefited from it [194]. Furthermore, FMT is associated with collateral side effects, namely abdominal discomfort, cramping, bloating, diarrhea, and constipation, and is also limited by donor requirements [24].

### 4.4. Nanoparticles and Microbiota

Nanoparticles are nanomaterials with three dimensions at the nanoscale. Due to their unique small size and high surface to volume ratio, they can reach the tumor site without being recognized by the immune system and protect their therapeutic cargo until reaching the tumor site. However, these promising features are also reported as being responsible for their eco and human toxicity [196].

Nanoparticles are broadly present in food, pharmacy, and medicine, and can directly impact the composition and/or metabolic activities of the gut microbiota [197,198]. Particularly, inorganic nanoparticles have been reported to be immunogenic and may affect the composition of the gut microbiota, potentially eliciting the development of chronic diseases and cancer [199,200,201,202].

Table 3 offers some examples of nanoparticles that can impact the gut microbiota.

Moreover, polymeric-based nanoparticles have also been implicated in gut microbiota dysfunction, such as poly(lactic-co-glycolic acid) (PLGA). Specifically, PLGA induces hepatic transcriptomic reprogramming in an obesity mouse model [217]. Moreover, a naturally occurring polymer, chitosan, with a broad spectrum of applications in nanomedicine formulations, has been reported to present prebiotic effects [218,219,220]. Interestingly, the use of *Lactobacillus acidophilus* ghosts (LAGs) has been studied for colon target therapies [221].

**Figure 7:**
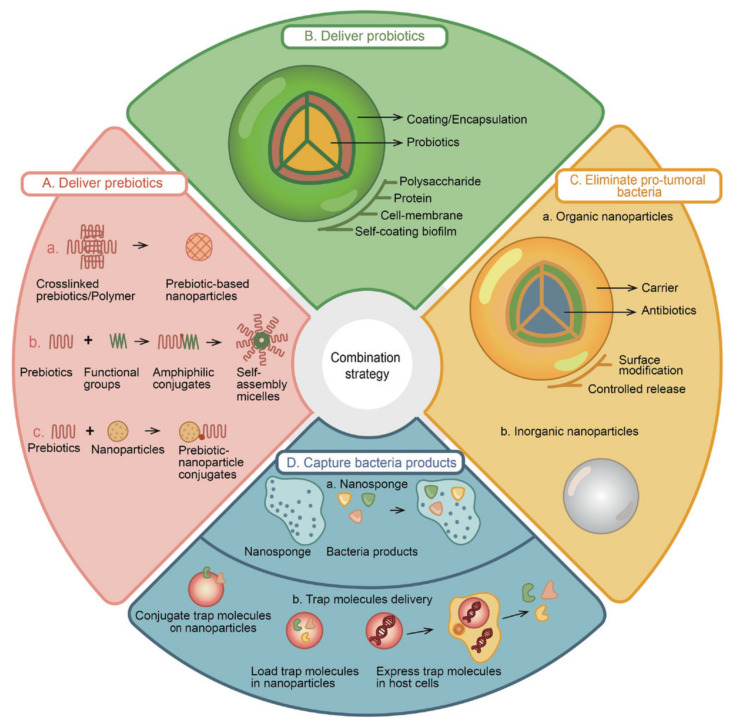
The modulation of microbiota by innovative drug delivery systems to improve cancer treatment outcomes. (**A**) Prebiotics can be applied in nanoparticles for drug delivery, conjugated with functional groups to form to nanoparticles alone or in combination with other cancer therapeutic agents. (**B**) Probiotics can be enclosed into nanoparticles to protect their bioactivities. (**C**) Eliminate pro-tumoral bacteria by targeted antibiotic delivery avoiding dysbiosis. (**D**) Nanoparticles can be designed to capture bacterial products or exhibit bacterial products inhibitors. Reprinted from [202] under a CC BY license.

The impact of the microbiota in carcinogenesis and the efficacy and modulation of cancer nanomedicines has captured scientific consideration. The use of drug delivery systems to modulate microbiota is summarized in Figure 7 [202].

Briefly, nanomedicine can work by eliminating cancer-causing bacteria, boosting probiotic bacteria, or the two strategies can be applied to restore the homeostasis of bacterial communities. Moreover, nanomedicine can target bacterial by-products. Different approaches, such as genetic engineering and synthetic biology, can promote the formulation of programmable bacterial devices [202]. Recently, the correlation and the inputs of nanoparticles and gut microbiota has been addressed for CRC [222]. 

Therefore, the symbiosis of nanomedicine and microbiota regulation may improve and catapult cancer treatment to a more specific and targetable paradigm, considering patient characteristics.

## 5. Conclusions

The human gut microbiota has been shipped for millions of years with impact in modulating host bodies. Technological development, particularly in bioinformatics and systems biology, has led to an extraordinary revolution in human knowledge and its interactions with microorganisms. The human organism presents a vast and complex network of interactions with the environment, including microorganisms. These multiple interactions contribute to maintaining its cellular homeostasis, whose alterations are connected with diseases, such as cancer. Identifying pro- or anti-tumorigenic species from complex oral and gut bacteria communities continues to be a tricky trail. Nonetheless, available human-based clinical and pre-clinical studies have demonstrated the interconnection of microbiota and digestive tract tumors through numerous mechanisms. The susceptibility of oral and intestinal microbiota to change in the face of pathogenic processes associated with protumoral processes and illness, and clinically used antitumor therapies have led to the establishment of putative microbiotic diagnostic and prognostic markers. Some of these studies have attempted to identify microbiotic risk factors and to evaluate their protective role against the side effects and toxicity of existing established antitumor therapies used in the clinical treatment of aerodigestive and digestive tumors. Additionally, the use of prophylactic and replacement therapy by bacteria dysregulated in digestive tumors is also being actively pursued. Besides probiotics, another critical tool widely investigated in re-establishing microbiota homeostasis and as adjuvant therapy in digestive tract tumors are the prebiotics and postbiotics, which have been indicated as key regulators of anticancer responses and immune modulation. Due to the complex interactions of individual bacterial strains with the integral biological system, tumors, and antitumor therapy, the choice of potential therapeutic pre-/pro- and/or postbiotic or their combination, has to be carefully considered. Therefore, the mechanisms and targets of their biological activity and their role in aerodigestive and digestive tumor pathologies should be extensively investigated before prophylactic/therapeutic/adjuvant application. Additionally, the selective and distinct biological activity of pre-/pro- and postbiotics might be another factor to consider when planning future therapeutic applications [223]. Recently, a novel application of intestinal microbes as delivery systems for CRC therapy was proposed. In this case, the probiotic-derived anticancer protein was delivered selectively to CRC cells in vivo [224]. Another factor that should be carefully considered is the use of animal in vivo models and their potential for translation into clinical practice. The interspecies differences in the native bacterial phyla and the concomitant physiological repercussions might be paramount for translating the results. Additionally, their susceptibility to maintain and reproduce the inoculated microbiota, such as in germ-free mice, is an important factor, especially when considering therapeutic approaches [225]. In this manuscript, some clues that can serve as compendium information were offered to contribute to decoding the debate between microbiota and the immune system and their interplay in aerodigestive and digestive cancer treatment outputs.

## Figures and Tables

**Table 1:** List of microbes designated as carcinogenic for humans according to the International Agency for Cancer Research (IACR) [25,26].

Microbe	Group	Cancer Type	References
Bacteria			
*Helicobacter pylori*	1	Non-Hodgkin lymphoma: all combined ^a^ Non-Hodgkin lymphoma: low-grade B-cell mucosa-associated lymphoid tissue (MALT) gastric lymphoma Stomach	[27,28,29,30]
Viruses			
Epstein–Barr virus	1	Burkitt lymphoma Hodgkin lymphoma Lymphoepithelioma-like carcinoma (LELC) ^a^ Non-Hodgkin lymphoma Non-Hodgkin lymphoma: extranodal NK/T cell lymphoma (nasal type) Non-Hodgkin lymphoma: immunosuppression-related lymphoma Pharynx: nasopharynx	[31,32,33,34]
Hepatitis B virus	1	Liver	[35,36]
Hepatitis C virus	1	Liver Non-Hodgkin lymphoma: all combined	[37] [38,39]
Human immunodeficiency virus type 1	1	Anus ^a^ Endothelium (Kaposi sarcoma) Eye Hodgkin lymphoma Liver ^a^ Non-Hodgkin lymphoma: all combined Skin ^a^ Uterine cervix	[40,41]
Human papillomavirus type 16	1	Penis Pharynx: oropharynx, tonsil Reto Uterine cervix Vagina Vulva	[42,43]
Human papillomavirus type 33	1	Anus ^a^	[44]
Human papillomavirus type 18	1	Anus ^a^ Oral cavity ^a^ Penis Uterine cervix	[45,46,47,48,49]
Human papillomavirus types 26, 53, 66, 67, 68, 70, 73, and 82	2B	Uterine cervix ^a^	[50,51,52]
Human papillomavirus types 31, 35, 39, 45, 51, 52, 56, 58, and 59	1	Uterine cervix	[53]
Human papillomavirus types 5 and 8	2B	Skin ^a^	[54]
Human T cell lymphotropic virus type 1	1	Adult T cell leukemia/lymphoma	[55]
Kaposi sarcoma herpesvirus	1	Endothelium (Kaposi sarcoma) Multicentric Castleman disease ^a^ Primary effusion lymphoma	[56]
Parasites			
*Clonorchis sinensis*	1	Bile duct	[57,58]
*Opisthorchis viverrini*	1	Bile duct	[59]
*Schistosoma haematobium*	1	Urinary bladder	[60]
*Schistosoma japonicum*	2B	Bile duct ^a^ Colon ^a^ Liver ^a^ Rectum ^a^	[61]

^a^, microbes that present limited evidence to induce cancer in humans.

**Table 2:** The influence of microbiota on the development of esophageal pathologies.

Samples	Method	Microbiota	References
Esophageal tumor and tumor-adjacent (A-ESCC) samples obtained from patients with esophageal squamous cell carcinoma (ESCC)	16S ribosomal RNA sequencing	56 taxa were detected with different intergroup distribution *R. mucilaginosa*, *P. endodontalis*, *N. subflava*, *H. pylori*, *A. parahaemolyticus*, and *A. rhizosphaerae* Enrichment of the species *P. endodontalis* and the reduction of *H. pylori* in tumor-adjacent tissues	[76]
Control vs. pathological esophagus	16S ribosomal ribonucleic acid V4 gene DNA sequencing	*Tissierella soehngenia*, and the genera: *Lactobacillus*, *Streptococcus*, *Acinetobacter*, and *Prevotella* are present on average in all samples Pathological esophagus showed significant decreases in the phylum *Planctomycetes* and *Crenarchaeota* compared with controls In BE samples with high-grade dysplasia, the presence of microorganisms of the genera *Nitrosopumilus*, *Balneola*, and *Planctomyces* was decreased	[77]
Normal squamous controls, non-dysplastic and dysplastic Barrett’s esophagus, and esophageal adenocarcinoma	16S rRNA gene amplicon sequencing	Decreased microbial diversity in esophageal adenocarcinoma tissue compared with healthy control tissues *Lactobacillus fermentum* was enriched in esophageal adenocarcinoma	[78]
ESCC and A-ESCC	16S rRNA	*Helicobacter pylori* infection may be an original cause of atypical hyperplasia of esophageal squamous epithelial tissues and contributed to the pathological carcinogenesis of ESCC.	[79]
Esophageal tissues from ESCC patients and normal controls	Immunohistochemistry 16S rDNA	*Porphyromonas gingivalis* is present in 61% of ESCC tissues vs. 12% in normal esophageal mucosa *P. gingivalis* infection could be a biomarker for the progression of ESCC	[80]
Tumor and non-tumor samples with ESCC or GCA	16S ribosomal RNA gene	Both tissue types are composed of *Firmicutes*, *Bacteroidetes*, and *Proteobacteria* ESCC tumor tissues contained more *Fusobacterium* and less *Streptococcus* than non-tumor tissues	[81]
Normal, esophagitis, or Barrett’s esophagus (intestinal metaplasia)	Bacterial 16S ribosomal RNA gene survey	Esophageal microbiomes can be divided into type I, where the genus *Streptococcus* is more abundant and typically concentrated in the normal esophagus, and into type II, where Gram-negative anaerobes/microaerophiles microbes are present and are primarily correlated with esophagitis and BE	[82]

**Table 3:** Examples of some in vivo studies using nanoparticles to address their impact on the gut microbiota.

Nanoparticles	Animal Model	Main Result	References
TiO_2_	Rats	↑ *Lactobacillus_reuteri* ↓ *Romboutsia* Hepatotoxicity	[203]
	Mice	↑ *Firmicutes* ↓ *Bacteroidetes* Intestinal mucus layer damage and dysbiosis	[204]
	Zebrafish	TiO_2_ and bisphenol induced ↑ *Lawsonia* ↓ *Hyphomicrobium*	[205]
	*Mytilus galloprovincialis*	↑ *Stenotrophomonas* ↓ *Shewanella, Kistimonas, Vibrio* TiO_2_ nanoparticles impact hemolymph microbiome composition that may result from the interplay between the microbiota and the immune system	[206]
	Mice	↓ *Bifidobacterium* and *Lactobacillus* Exacerbated immune responses in vivo	[207]
	Mice	↓ *Bifidobacterium* Prebiotic inulin supplementation prevented TiO_2_ nanoparticles-induced colonic barrier dysfunction	[208]
	White albino mice	TiO_2_ from chocolates inhibited the growth and activity of *Bacillus coagulans*, *Enterococcus faecalis*, and *Enterococcus faecium*	[209]
AgNPs	Sprague Dawley rats	Ileal mucosal microbial populations, alterations apparent ↓ in *Firmicutes* phyla ↓ expression of important immunomodulatory genes, including MUC3, TLR2, TLR4, GPR43, and FOXP3	[210]
	*Spodoptera litura*	↓ *Klebsiella pneumoniae, Bacillus licheniformis,* and *Bacillus cereus* and *Citrobacter freundi, Enterobacter cloacae*	[211]
	*Drosophila melanogaster*	↓ in the diversity ↑ *Lactobacillus brevis* ↓ *Acetobacter*	[212]
ZnNPs	Chicken	↑ *Bacteroides* and *Faecalibacterium* ↓ *Lactobacillus*	[213]
SiO_2_	Mice	↑ *Bacteroidetes* ↓ *Firmicutes*	[214]
Iron(III) oxo-hydroxidenano	Rats	Fe(III) supplemented group ↑ *Lactobacillus* spp. ↓ *Bacteroides* spp. Compared with animals supplemented with Fe(II) sulfate	[215]
CuNPs	*Danio rerio*	Suppression in beneficial bacteria *Cetobacterium somerae*	[216]

## Data Availability

Not applicable.
